# Effect of *Aspergillus* and *Bacillus* Concentration on Cotton Growth Promotion

**DOI:** 10.3389/fmicb.2021.737385

**Published:** 2021-10-13

**Authors:** Paola Andrea Escobar Diaz, Roberta Mendes dos Santos, Noemi Carla Baron, Oniel Jeremias Aguirre Gil, Everlon Cid Rigobelo

**Affiliations:** Laboratory of Soil Microbiology, Faculty of Agricultural and Veterinary Sciences, Department of Agricultural Production Sciences, São Paulo State University, São Paulo, Brazil

**Keywords:** rhizobacteria, *Aspergillus sydowii*, *Bacillus* sp., yield, growth promoters, inoculants

## Abstract

There are no studies in literature on the effect of inoculant concentrations on plant growth promotion. Therefore, in the present study, two experiments were carried out, one under pot conditions and the other in the field with cotton crop, in order to verify the effect of *Aspergillus* and *Bacillus* concentrations on the biometric and nutritional parameters of plant and soil, in addition to yield. The pot experiment evaluated the effect of different concentrations, ranging from 1 × 10^4^ to 1 × 10^10^ colony-forming units per milliliter (CFU mL^–1^) of microorganisms *Bacillus velezensis* (Bv188), *Bacillus subtilis* (Bs248), *B. subtilis* (Bs290), *Aspergillus brasiliensis* (F111), *Aspergillus sydowii* (F112), and *Aspergillus* sp. *versicolor* section (F113) on parameters plant growth promotion and physicochemical and microbiological of characteristics soil. Results indicated that the different parameters analyzed are influenced by the isolate and microbial concentrations in a different way and allowed the selection of four microorganisms (Bs248, Bv188, F112, and F113) and two concentrations (1 × 10^4^ and 1 × 10^10^ CFU mL^–1^), which were evaluated in the field to determine their effect on yield. The results show that, regardless of isolate, inoculant concentrations promoted the same fiber and seed cotton yield. These results suggest that lower inoculant concentrations may be able to increase cotton yield, eliminating the need to use concentrated inoculants with high production cost.

## Introduction

The use of plant-growth promoting microorganisms (PGPMs) has increased in the world as an alternative to the excessive application of mineral fertilizers that can contribute to soil degradation, emission of polluting gases into the atmosphere, and reduction of biodiversity in different ecosystems (Singh et al., 2016).

Inoculants are products that have in their composition live microorganisms capable of promoting plant development with different mechanisms or modes of action, such as production of phytohormones and siderophores, phosphate solubilization, and induction of resistance against abiotic and biotic stresses (Bhattacharyya and Jha, 2012; Malusá and Vassilev, 2014). PGPM application has been carried out in several agricultural cultures, and many studies have been developed to elucidate its mode of action in plants to meet the new requirements of industries in the sector and agricultural producers. The microorganisms most frequently used as inoculants are fungi of the genera *Trichoderma*, *Purpureocillium*, *Metarhizium*, *Beauveria*, and *Aspergillus* (Behie and Bidochka, 2014; Samson et al., 2014; Alori and Babalola, 2018; Baron et al., 2018, 2020; Ahmad et al., 2020), and bacteria of the genera *Azospirillum*, *Azotobacter*, *Bacillus*, *Enterobacter*, and *Streptomyces* (Kloepper et al., 1989; Okon and Labandera-Gonzalez, 1994; Glick et al., 1999; Tahmatsidou et al., 2006; Marulanda et al., 2009; Pedraza et al., 2010; Diaz et al., 2019).

Under field conditions, PGPMs are applied in the form of formulated products, which contain inerts and additives in addition to the active ingredient, which is the microorganism. The search for new inoculant formulations, which enhance plant development in order to reduce the use of mineral fertilizers, thus contributing to more sustainable agriculture, is increasing (Malusá and Vassilev, 2014; Bizos et al., 2020). These new formulations have included increasing the concentration of microorganisms to be applied in the field. However, despite the advance in the use of inoculants in agriculture, there are few studies that have evaluated the effect of inoculant concentration on plant growth promotion, particularly in cotton. Thus, this theme has become essential to define whether the increase in the concentration of microorganisms is an important aspect related to product efficiency or whether it is just an aspect of commercial advantage.

In this study, cotton was used because it is a crop that stands out for its high demand for mineral fertilizers and phytosanitary products to ensure good productivity, a situation that causes serious changes in the environment (Michereff and Barros, 2001; Carvalho and Barcellos, 2012).

The aim was to determine the effect of different concentrations of microorganisms *Bacillus velezensis*, *Bacillus subtilis*, *Aspergillus brasiliensis*, *Aspergillus sydowii*, and *Aspergillus* sp. (*versicolor* section) on the growth of cotton plants under pot conditions in greenhouse and field conditions.

## Materials and Methods

### Study Location

According to the Köppen and Geiger classification, the climate of the region corresponds to a tropical climate with dry season in the winter (Peel et al., 2007). The predominant soil at the site is classified as Red Eutrophic Latosol (Oxisol) with clayey texture (52% clay, 23% silt, and 24% total sand) (EMBRAPA, 2006).

### Experiment 1: Determination of the Effect of Inoculation of Microorganisms at Different Concentrations in Greenhouse

#### Microorganisms and Inoculant Preparation

Microorganisms (bacteria and fungi) used in this study belong to the collection of the Laboratory of Soil Microbiology, UNESP, Campus of Jaboticabal (Table 1) and were selected for presenting growth-promoting characteristics such as phosphorus solubilization, biological nitrogen fixation, and indole acetic acid production (Baron et al., 2018; Diaz et al., 2019; Milani et al., 2019).

**TABLE 1:** Description of microorganisms.

Microorganisms	Code in the collection	GenBank deposit number
*Bacillus subtilis*	Bs248	MZ133755
*B. subtilis*	Bs290	MZ133476
*Bacillus velezensis*	Bv188	MZ133757
*Aspergillus brasiliensis*	F111	MZ133758
*Aspergillus sydowii*	F112	MZ133759
*Aspergillus* sp. (*versicolor* section)	F113	MZ133456
Control	–	–

The microorganisms used in the study were pre-inoculated in Petri dishes containing nutrient agar for bacteria and potato dextrose agar for fungi. Incubation was carried out in BOD oven at 30°C for 24 h for bacteria and at 25°C for 7 days for fungi.

Each bacterial isolate was multiplied in Erlenmeyer flask containing 90 ml of sterile nutrient broth medium inoculated with isolates prepared on Petri dishes. Flasks were incubated at 30°C for 24 h under agitation at 150 rpm. Then, absorbance readings of each isolate were carried out in spectrophotometer at 600 nm to determine the optical density. In addition, 100 μl of each flask with the different isolates was seeded in Petri dishes containing nutrient agar for the determination and adjustment of cell concentrations (Kloepper et al., 1989).

For fungi, conidium suspension was prepared by scraping Petri dishes containing mycelium cultivated on potato dextrose agar for 7–10 days at 25°C. For scraping, 0.1% Tween 80 solution was used. Fungi suspensions obtained were filtered in sterile voile to remove excess mycelium. The determination of the conidium concentration of each fungus was performed by counting in Neubauer chamber. For all microorganisms (bacteria and fungi), concentrations of 1 × 10^4^, 1 × 10^6^, 1 × 10^8^, and 1 × 10^10^ colony-forming units/ml (CFU mL^–1^) were standardized for bacteria and conidia ml for fungi.

### Seed Inoculation

Cotton seeds were individually inoculated with microorganisms (bacteria or fungi) by immersion for 8 h at 25°C (Jaber and Enkerli, 2016). Immersion was carried out in the dark under agitation at 130 rpm. This procedure was performed for all microorganisms and concentrations. After the immersion period, cotton seeds were sown in pots containing previously sieved soil.

Cotton seedlings were inoculated three times from the beginning to the end of the experiment at 15-day intervals. In each inoculation, 10 ml of suspension containing the respective microorganism at concentrations of 1 × 10^4^, 1 × 10^6^, 1 × 10^8^, and 1 × 10^10^ CFU mL^–1^ for bacteria and conidia ml for fungi was applied per pot. Inoculations were performed by applying the inoculum at the base and stem of plants using graduated micropipette (Kasvi monocanal premium black k1-1000 PB).

### Experimental Design and Experiment Management

The experiment was carried out at the Horticulture Sector of the “Júlio de Mesquita Filho” São Paulo State University (UNESP), Campus of Jaboticabal, São Paulo, Brazil. The experiment was arranged in a randomized block design with 6 × 4 factorial arrangement + 1 additional treatment (control) with five replicates, totaling 125 pots. Microorganism factor sublevels were Bs248, Bs290, Bv188, F111, F112, and F113 (Table 1). Concentration factor sublevels were 1 × 10^4^, 1 × 10^6^, 1 × 10^8^, and 1 × 10^10^ CFU or conidia ml^–1^. Pots of 5-L capacity were filled with sieved soil (particles smaller than 1 cm in diameter) and fertilized according to previously performed soil analysis (Table 2) and nutritional recommendations for pot experiments proposed by Malavolta et al. (1997) for cotton crop. Nitrogen (N: 3.33 g urea/pot), phosphorus (P: 5.5 g P_2_O_5_/pot), potassium (K: 1.66 g KCl/pot), calcium (Ca: 6.25 g super single/pot), magnesium (Mg: 0.5 g MgO/pot), sulfur (S: 3.125 g super single/pot), zinc (Zn: 0.125 g ZnSO_4_/pot), boron (B: 0.025 g H_3_BO_3_/pot), molybdenum (Mo: 0.002 g molybdate/pot), copper (Cu: 0.03 g CuSO_4_/pot), and manganese (Mn: 0.08 g MnSO_4_/pot) were added. All nutrients were mixed with the sieved soil 1 week before sowing. The moisture content of pots was kept around 70% of the field capacity with daily irrigations.

**TABLE 2:** Analysis of soil used in greenhouse and field experiments.

pH	OM	P	K	Ca	Mg	H + Al	S.B.	CEC	V
CaCl_2_	g/dm^3^	Mg/dm^3^	……………………mmol_c_/dm^3^…………………						%
6.9	10	23	0.7	79	13	11	93.4	104.2	90

*OM, organic matter; S.B., Ca + Mg + Na + K; CEC, S.B. + H + Al; V%, (S.B./CEC) ^∗^ 100.*

Five cotton seeds (*Gossypium hirsutum*–IMA7501 WS) were sown per pot; and 15 days after seedling emergence, thinning was performed, keeping one plant per pot. The experiment was carried out until the flowering of cotton plants, 70 days after emergence.

### Evaluated Parameters

#### Shoot and Root Dry Matter

Plants were collected and separated into shoots and roots, washed in running water, and placed in paper bags for drying in oven with air circulation at 65°C until reaching constant weight. Root and shoot dry matter weight was determined using analytical scale.

#### Preparation of Soil Samples

Samples were separated into two subsamples of approximately 100 g each. A subsample was sieved and dried at room temperature for chemical analysis, and the other was kept in a refrigerator for microbiological analysis.

#### Counting Bacteria Present in the Soil

Ten grams of soil was placed in an Erlenmeyer flask containing 95 ml of 0.1% sodium pyrophosphate saline solution. All Erlenmeyer flasks were shaken for 1 h at 130 rpm, and the contents of flasks were used to prepare serial dilutions following methodology proposed by Wollum (1982). Aliquots of 100 μl of obtained dilutions were inoculated into Petri dishes containing nutrient agar medium or potato dextrose agar in triplicate. Plates were kept in BOD oven at 30°C for bacteria and 25°C for fungi. The number of CFU mL^–1^ was verified after 24, 48, and 72 h (Vieira and Nahas, 2000).

#### Counting of Endophytic Bacteria and Fungi

Plants were separated into leaves and roots and washed with running water. Samples containing 3 g of each vegetative tissue (leaves and roots) were submitted to superficial disinfection to eliminate epiphytic microorganisms. Each tissue (leaf or root) was sequentially immersed in 70% ethanol for 1 min, sodium hypochlorite solution (2.0–2.5% active Cl) for 4 min, and 70% ethanol for 30 s. Subsequently, tissues were washed three times with distilled water. Once washed and disinfected, tissues were macerated with 3 ml of sterile 0.85% saline solution with the aid of a flask and a pestle (de Araújo et al., 2002). The macerated material was used to prepare serial dilutions, and 100 μl of aliquots was seeded in Petri dishes containing tryptone soy agar (TSA) medium for bacterial isolation and potato dextrose agar for fungal isolation. Plates were grown in microbiological greenhouses at constant temperature of 30°C for 24 h for bacterial growth and at 25°C for 7 days in the case of fungal isolation (Caruso et al., 2000). Microorganism counts were performed in separate groups, fungi, and bacteria with their respective controls.

#### Determination of the Phosphorus Concentration in Plants and Soil

The determination of soluble soil phosphorus was carried out using the method proposed by Watanabe and Olsen (1965). For the determination of phosphorus in plants, phosphorus concentrations in roots and shoots were determined according to methodology proposed by Haag et al. (1975) and modified by Bezerra Neto and Barreto (2011).

#### Determination of the Total Nitrogen Concentration in Plants and Soil

The determination of the nitrogen concentration in shoots and roots was performed according to Haag et al. (1975) with sulfuric digestion of plant material to estimate the nitrogen concentration or dose associated with obtaining 90% of dry matter production. For the determination of total nitrogen in soil, the methodology proposed by Bremner and Mulvaney (1983) and modified by Wilke (2005) was used.

#### Microbial Respiratory Activity

The respiratory activity was determined by the method of quantification of released CO_2_ according to Jenkinson and Powlson (1976), using wide-mouth flasks with 100 g of soil (dry or wet). Inside flasks, two beakers (one containing 20 ml of NaOH, and the other 20 ml distilled water) were placed, were then sealed with plastic film, and incubated in the dark for 7 days. Microbial respiration was estimated from the amount of CO_2_ released from soil samples in a continuous air flow system free from CO_2_ and moisture. After incubation, the remaining NaOH was quantified by titration with HCl.

#### Microbial Biomass Carbon

Microbial biomass carbon was determined by the irradiation-extraction method (Islam and Weil, 1998; Mendonça and Matos, 2017), using microwave oven. After irradiation, samples were submitted to 0.5 mol/L of potassium sulfate extractor, and microbial biomass carbon was determined by oxidation with 0.066 mol/L of potassium dichromate followed by titration with 0.033 mol/L of ammonia ferrous sulfate (Brookes et al., 1982).

### Statistical Analysis

Prior to analysis of variance, data normality (the Kolmogorov–Smirnov test) and homogeneity of variances (Levene’s test) were tested for each parameter evaluated. Data were transformed into (x + 0.5)^1/2^ to comply with assumptions of the analysis of variance. Comparisons of means were performed using Tukey’s test (α ≤ 0.05). Analyses were performed using the R 3.4.1 open software for Windows (R Core Team, 2020).

### Experiment 2: Determination of the Effect of Inoculation of Microorganisms on Cotton Plants Under Field Conditions

#### Cotton Planting

The experiment was carried out at the Teaching, Research and Extension Farm (FEPE) – UNESP, Jaboticabal, São Paulo, during the off season (January–June 2020). The field soil was classified as Red Eutrophic Latosol (Oxisol) with clayey texture. Soil chemical analysis is detailed in Table 2.

Soil fertilization was performed once before sowing using the 8–28–16 of NPK + 0.5% Zn formula, with the amount of nitrogen 80% lower than the requirement to avoid masking the effect produced by microorganisms and their concentrations on cotton yield. Cotton was sown at spacing of 1 m between rows and 8–10 seeds per linear meter. The dimensions of the plot were 5 m in length by 5 m in width with useful area of 15 m^2^.

The microorganisms used in the experiment were selected based on results of experiment 1. Microorganisms Bs248, Bv188, F112, and F113 were tested at concentrations of 1 × 10^4^ and 1 × 10^10^ CFU or conidia ml^–1^. The multiplication of these microorganisms was performed as previously described in experiment 1. Application was performed three times, every 15 days, using back sprayer with constant pressure. In this experiment, seeds were not inoculated, and the first application was carried out 7 days after the emergence of cotton seedlings.

Microorganisms were applied at dose of 1 L of suspension per hectare (ha). The amount of water used was 200 L/ha (500 ml per useful area of 15 m^2^). The control treatment was sprayed with water only. Cotton was manually harvested 151 days after seedling emergence. Seed cotton was harvested from plants of the useful plot (15 m^2^).

#### Experimental Design and Experiment Management

A randomized block design with 4 × 2 factorial arrangement + 1 additional treatment (control) with four replicates was used. Microorganism factor sublevels were Bs248, Bv188, F112, and F113. Concentration factor sublevels were 1 × 10^10^ and 1 × 10^4^ CFU mL^–1^. Crop management was carried out considering commercial management for the region.

#### Evaluated Parameters

Parameters were evaluated by manual harvesting of plants in useful plots. The weight of seed cotton was measured using analytical scale. After drying in oven with air circulation at 65°C, seeds were manually separated from fibers and weighed on analytical scale. Fiber weight was obtained by the difference between the weight of the cotton harvested and the weight of the seed. Seed weight and fiber weight were estimated in kg/ha.

#### Data Analysis

Analyses were performed using the R software for Windows (R Core Team, 2020). The normality and homogeneity of variances were assessed using the Shapiro–Wilk test and Levene’s test (α ≤ 0.05), respectively. Treatments were analyzed using ANOVA, followed by Tukey’s test (α ≤ 0.05) to compare the mean of treatments.

## Results and Discussion

### Experiment 1: Determination of the Effect of Inoculation of Microorganisms at Different Concentrations in Greenhouse

**FIGURE 1:**
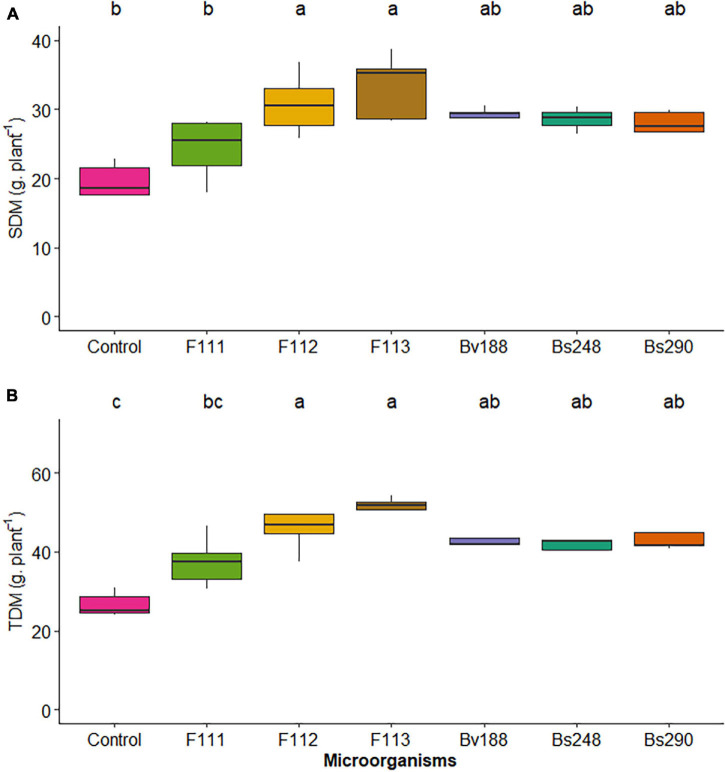
Boxplots (median and quartiles) of SDM **(A)** and TDM **(B)** in cotton inoculated with plant growth-promoting microorganisms. Different lowercase letters in the line indicate statistical difference between means (Tukey, *p* < 0.05). F111, *Aspergillus brasiliensis*; F112, *Aspergillus sydowii*; F113, *Aspergillus* sp.; Bv188, *Bacillus velezensis* strain Bv188; Bs248, *Bacillus subtilis* strain Bs248; Bs290, *B. subtilis* strain Bs290; Ctrl, control; SDM, shoot dry matter; TDM, total dry matter.

The results indicate that there was no interaction between microorganism factor and inoculant concentration for variables shoot, root, and total dry matter in cotton plants. This means that regardless of microorganism, the behavior was the same, given the different inoculant concentrations. Furthermore, there was no effect of the concentration factor on variables shoot, root and total dry matter, nitrogen content in root dry matter, phosphorus in shoot dry matter, and biomass carbon; however, there was a significant effect of the microorganism factor on variables shoot (Figure 1A) and total (Figure 1B) dry matter, highlighting fungi *A. sydowii* and *Aspergillus* sp. *versicolor* section, with values of 30.83 and 33.40 g/plant, respectively, for shoot dry matter, and 47.71 and 51.20 g/plant, respectively, for total dry matter, compared with control treatment, which was 23.40 g/plant for shoot dry matter and 30.04 g/plant for total dry matter.

Plant–fungus associations are mainly established by two groups of fungi, mycorrhizal and endophytic fungi (Bonfante and Genre, 2010). Endophytic fungi are those capable of living endosymbiotically with plants without causing disease symptoms (Behie and Bidochka, 2014). They can act as plant growth promoters, increase germination rate, improve seedling establishment, and increase plant resistance to biotic and abiotic stresses, producing antimicrobial compounds, phytohormones, and other bioactive compounds. In addition, endophytic fungi are responsible for the acquisition of soil nutrients, including macronutrients such as phosphorus, nitrogen, potassium, and magnesium, and micronutrients such as zinc, iron, and copper (Behie and Bidochka, 2014; Rai et al., 2014; Khan et al., 2015).

Soil fungi are widely distributed and participate in ecological processes that influence plant growth and soil health. It is considered that the diversity of fungi that inhabit the soil and the rhizosphere can reach more than 200 species in a single soil (Vandenkoornhuyse et al., 2002).

Several *Aspergillus* species are commercially exploited due to their ability to produce and secrete many enzymes and metabolites, such as antibiotics and mycotoxins (Volke-Sepulveda et al., 2016). The ability of fungi of the genus *Aspergillus* to produce secondary metabolites is very important because they play a vital role in survival and adaptation in soil; in addition, they are involved in the degradation of a wide range of natural organic substrates, particularly plant materials (Goldman and Osmani, 2008).

**FIGURE 2:**
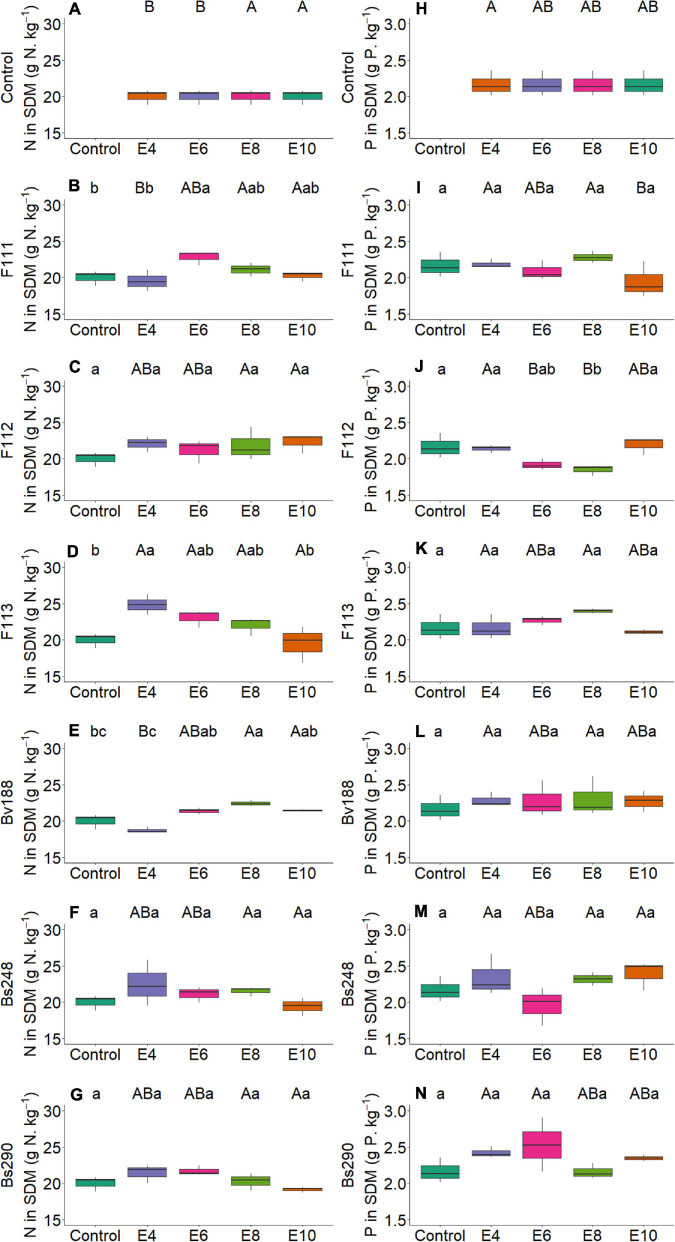
Boxplots (median and quartiles) of nitrogen **(A–G)** and phosphorus **(H–N)** content in SDM in cotton inoculated with plant growth-promoting microorganisms. Different lowercase letters in a row and uppercase letters in a column indicate statistical difference between means (Tukey, *p* < 0.05). F111, *Aspergillus brasiliensis*; F112, *Aspergillus sydowii*; F113, *Aspergillus* sp.; Bv188, *Bacillus velezensis* strain Bv188; Bs248, *Bacillus subtilis* strain Bs248; Bs290, *B. subtilis* strain Bs290; E4, 1 × 10^4^; E6, 1 × 10^6^; E8, 1 × 10^8^; E10, 1 × 10^10^ conidia or CFU mL^−1^; Ctrl, control; SDM, shoot dry matter.

**FIGURE 3:**
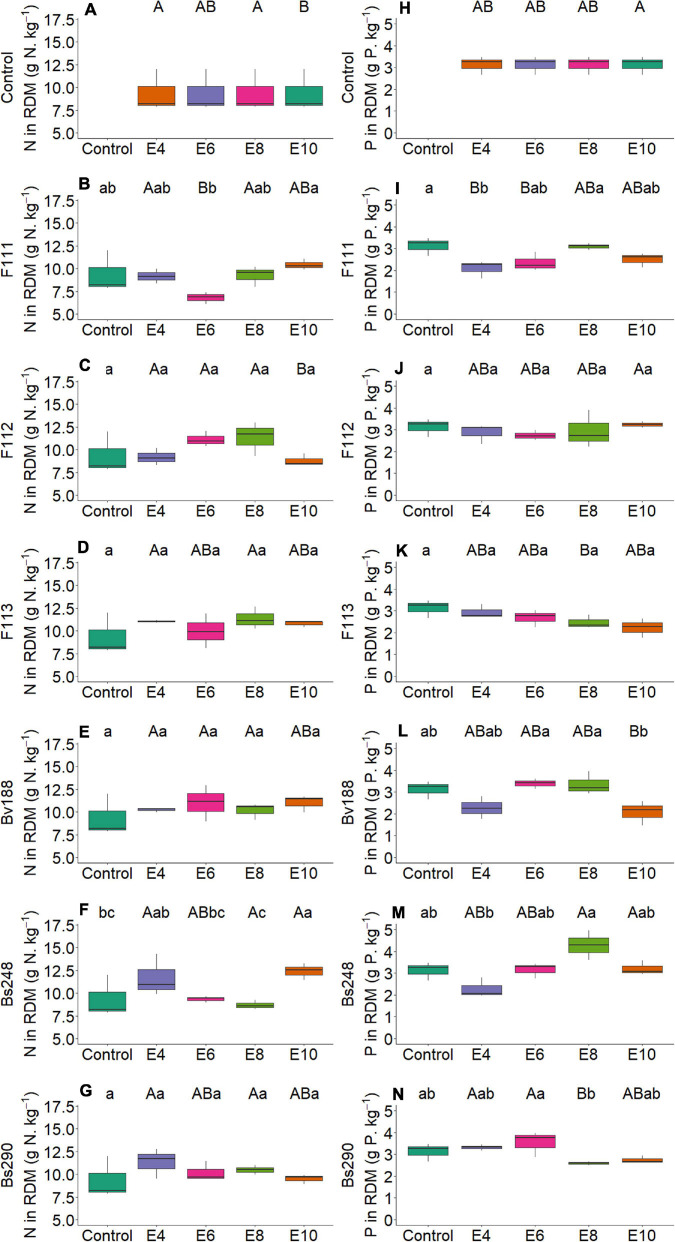
Boxplots (median and quartiles) of nitrogen **(A–G)** and phosphorus **(H–N)** content in RDM in cotton inoculated with plant growth-promoting microorganisms. Different lowercase letters in a row and uppercase letters in a column indicate statistical difference between means (Tukey, *p* < 0.05). F111, *Aspergillus brasiliensis*; F112, *Aspergillus sydowii*; F113, *Aspergillus* sp.; Bv188, *Bacillus velezensis* strain Bv188; Bs248, *Bacillus subtilis* strain Bs248; Bs290, *B. subtilis* strain Bs290; E4, 1 × 10^4^; E6, 1 × 10^6^; E8, 1 × 10^8^; E10, 1 × 10^10^ conidia or CFU mL^−1^; Ctrl, control; RDM, root dry matter; CFU, colony-forming units.

**FIGURE 4:**
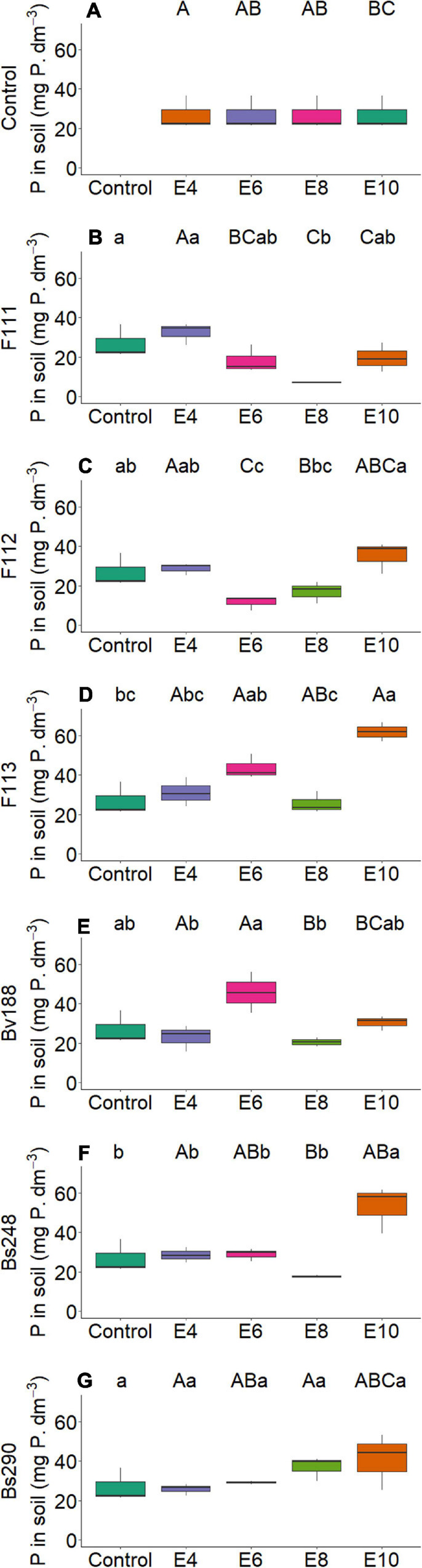
Boxplots (median and quartiles) of phosphorus in soil sown with cotton and inoculated with plant growth-promoting microorganisms: Control **(A)**; F111 **(B)**; F112 **(C)**; F113 **(D)**; Bv188 **(E)**; Bs248 **(F)**; and Bs290 **(G)**. Different lowercase letters in row and uppercase letters in column indicate statistical difference between the means (Tukey, *P* < 0.05). Abbreviations: F111, *Aspergillus brasiliensis*; F112, *A. sydowii*; F113, *Aspergillus* sp.; Bv188, *B. velezensis* strain Bv188; Bs248, *B. subtilis* strain Bs248; Bs290, *B. subtilis* strain Bs290; E4, 1 × 10^4^; E6, 1 × 10^6^, E8, 1 × 10^8^; E10, 1 × 10^10^ conidia or CFU/ml; Ctrl, Control; CFU, colony- forming units.

**FIGURE 5:**
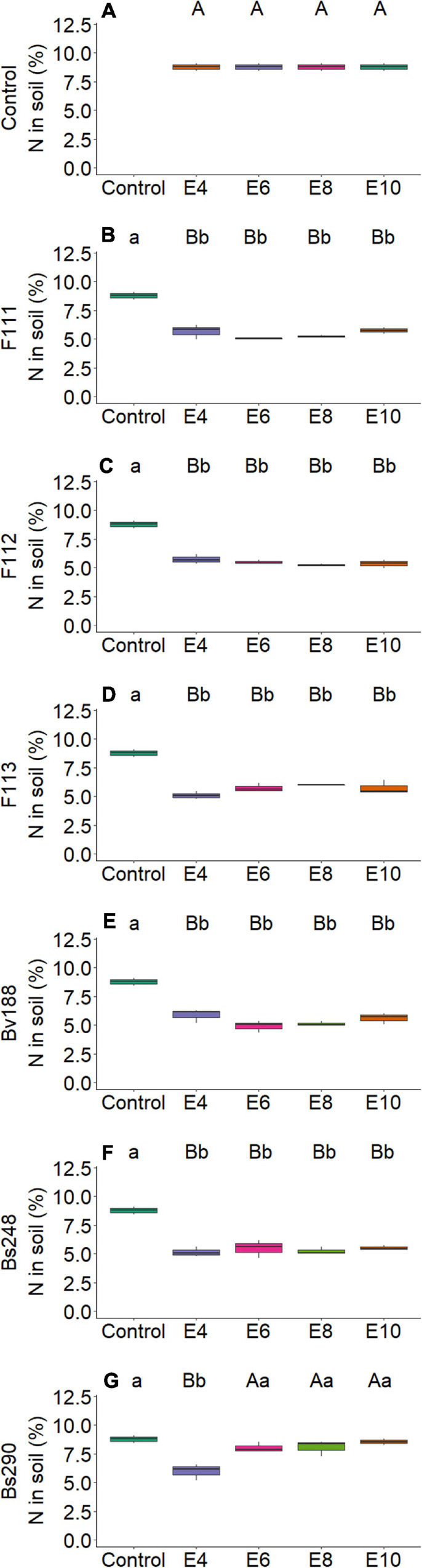
Boxplots (median and quartiles) of percentage of nitrogen in soil sown with cotton and inoculated with plant growth-promoting microorganisms: Control **(A)**; F111 **(B)**; F112 **(C)**; F113 **(D)**; Bv188 **(E)**; Bs248 **(F)**; and Bs290 **(G)**. Different lowercase letters in row and uppercase letters in column indicate statistical difference between means (Tukey, *P* < 0.05). Abbreviations: F111, *Aspergillus brasiliensis*; F112, *A. sydowii*; F113, *Aspergillus* sp.; Bv188, *B. velezensis* strain Bv188; Bs248, *B. subtilis* strain Bs248; Bs290, *B. subtilis* strain Bs290; E4, 1 × 10^4^; E6, 1 × 10^6^; E8, 1 × 10^8^; E10, 1 × 10^10^ conidia or CFU/ml; Ctrl, Control; CFU, colony- forming units.

**FIGURE 6:**
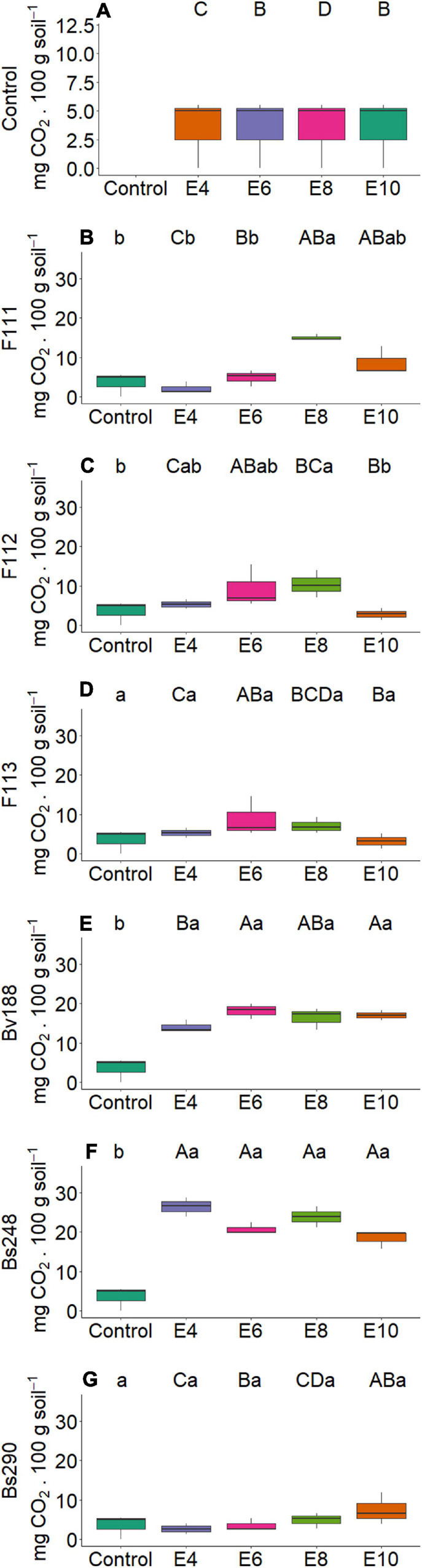
Boxplots (median and quartiles) of respiratory activity in soil sown with cotton and inoculated with plant growth-promoting microorganisms: Control **(A)**; F111 **(B)**; F112 **(C)**; F113 **(D)**; Bv188 **(E)**; Bs248 **(F)**; and Bs290 **(G)**. Different lowercase letters in row and uppercase letters in column indicate statistical difference between means (Tukey, *P* < 0.05). Abbreviations: F111, *Aspergillus brasiliensis*; F112, *A. sydowii*; F113, *Aspergillus* sp.; Bv188, *B. velezensis* strain Bv188; Bs248, *B. subtilis* strain Bs248; Bs290, *B. subtilis* strain Bs290; E4, 1 × 10^4^; E6, 1 × 10^6^; E8, 1 × 10^8^; E10, 1 × 10^10^ conidia or CFU/ml; Ctrl, Control; CFU, colony- forming units.

**FIGURE 7:**
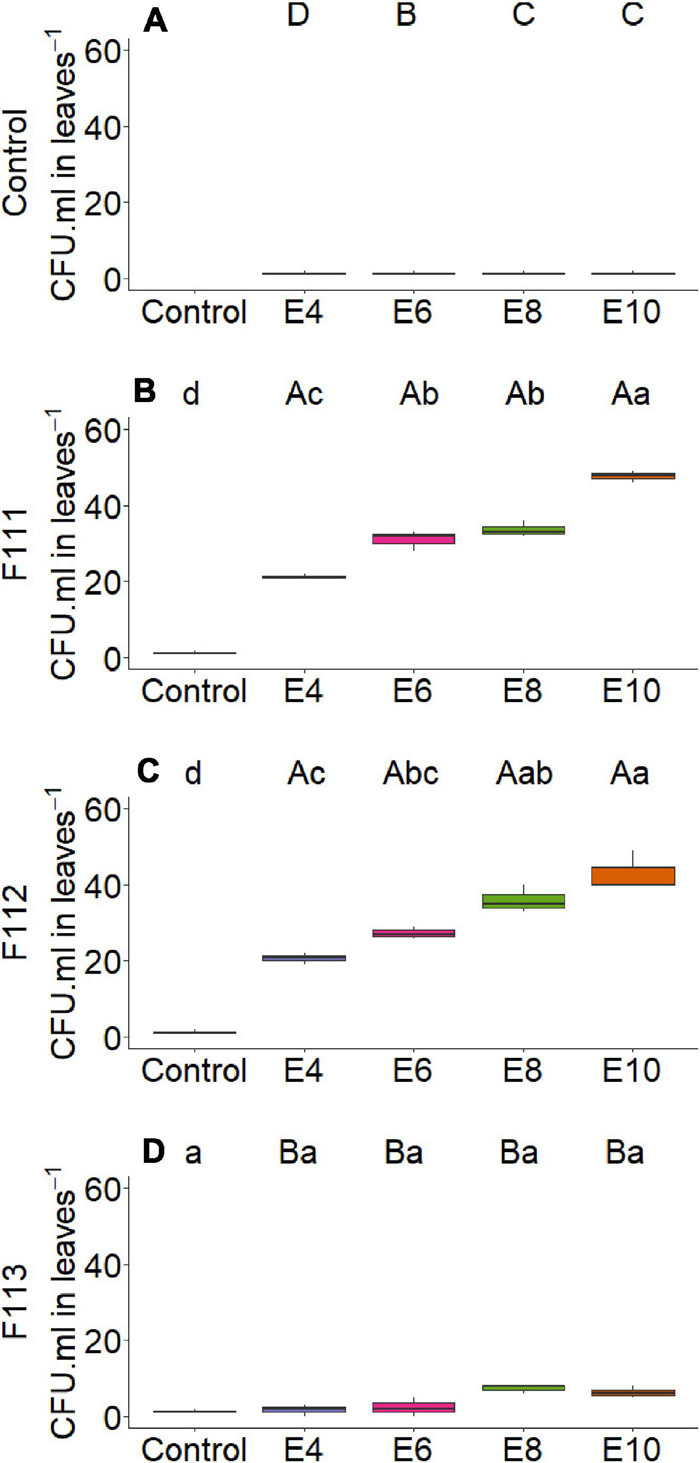
Boxplots (median and quartiles) of CFU in cotton leaves inoculated with **(A)** *Aspergillus brasiliensis* **(B)**, *Aspergillus sydowii* **(C)**, and *Aspergillus* sp. **(D)** in four concentrations. Different lowercase letters in a row and uppercase letters in a column indicate statistical difference between means (Tukey, *p* < 0.05). F111, *Aspergillus brasiliensis*; F112, *A. sydowii*; F113, *Aspergillus* sp.; E4, 1 × 10^4^; E6, 1 × 10^6^; E8, 1 × 10^8^; E10, 1 × 10^10^ conidia or CFU mL^−1^; Ctrl, control; and CFU, colony-forming units.

**FIGURE 8:**
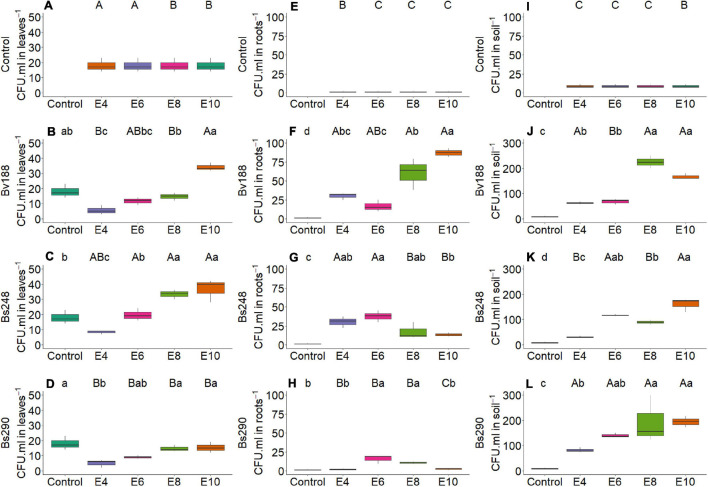
Boxplots (median and quartiles) of CFU in leaves **(A–D)**, root **(E–H)**, and soil **(I–L)** inoculated with plant growth-promoting microorganisms. Different lowercase letters in a row and uppercase letters in the vertical indicate statistical difference between means (Tukey, *p* < 0.05). Bv188, *Bacillus velezensis* strain Bv188; Bs248, *Bacillus subtilis* strain Bs248; Bs290, *B. subtilis* strain Bs290; E4, 1 × 10^4^; E6, 1 × 10^6^; E8, 1 × 10^8^; E10, 1 × 10^10^ conidia or CFU mL^−1^; Ctrl, control; CFU, colony-forming units.

**FIGURE 9:**
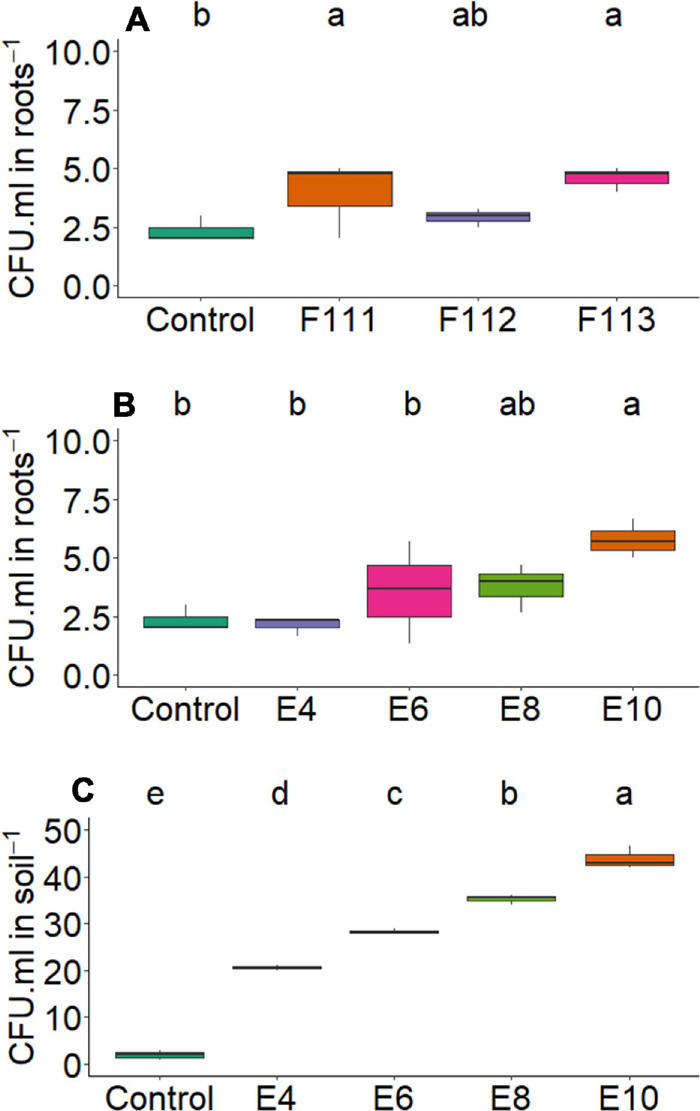
Boxplots (median and quartiles) of CFU in root **(A,B)** and soil **(C)** inoculated with *Aspergillus brasiliensis*, *Aspergillus sydowii*, and *Aspergillus* sp. Different lowercase letters in a row indicate statistical difference between means (Tukey, *p* < 0.05). F111, *Aspergillus brasiliensis*; F112, *A. sydowii*; F113, *Aspergillus* sp.; E4, 1 × 10^4^; E6, 1 × 10^6^; E8, 1 × 10^8^; E10, 1 × 10^10^ conidia or CFU mL^−1^; Ctrl, control; CFU, colony-forming units.

On the other hand, there was interaction between microorganism factor and inoculant concentration with variables nitrogen and phosphorus content in shoot (Figure 2) and root dry matter (Figure 3), soil phosphorus (Figure 4), soil nitrogen percentage (Figure 5), respiratory activity (Figure 6), colony-forming units in leaves (Figures 7, 8), and colony-forming units in roots and soil (Figure 9).

For fungus *A. brasiliensis*, the unfolding of interactions indicates that inoculation in cotton plants at a concentration of 1 × 10^6^ conidia ml^–1^ favored the increase in shoot nitrogen content (22.75 g N/kg; Figure 2B); root and soil phosphorus contents were lower at concentrations of 1 × 10^4^ and 1 × 10^8^ conidia ml^–1^, with values of 2.09 g P/kg and 7.10 mg P/dm^3^ soil, when compared with controls (3.13 g P/kg and 26.91 mg P/dm^3^ soil, respectively) (Figures 3I, 4B). Species of the genus *Aspergillus*, according to Souchie et al. (2006), Pacheco and Damasio (2013), and de Oliveira Mendes et al. (2014), highlight the phosphorus solubilization capacity and its potential for use as solubilizers for different sources of phosphorus in the soil. Schneider et al. (2010) reported the ability to synthesize organic acids and produce large amounts of citric acid, which is one of the main factors responsible for the solubilization of phosphorus in these fungi. The soil nitrogen percentage was lower than that of control at all inoculant concentrations (Figure 5B). These results suggest that *A. brasiliensis* can serve as hosts for nitrogen-fixing bacteria (endosymbionts) (Paul et al., 2020). These interactions may allow the plant to have absorbed nitrogen fixed and/or contained in the soil. The nitrogen-fixing property is absent in eukaryotes, but they circumvented this deficiency by associating with nitrogen-fixing bacteria (Kneip et al., 2007).

**FIGURE 10:**
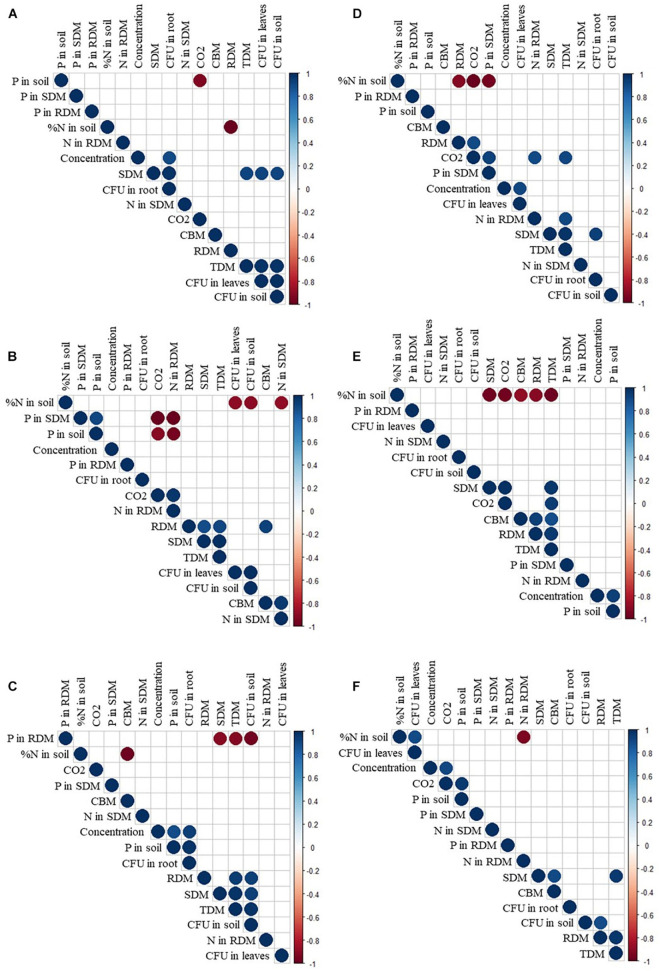
Correlation of growth promotion variables and concentration of *Aspergillus brasiliensis* **(A)**, *Aspergillus sydowii* **(B)**, *Aspergillus* sp. **(C)**, *Bacillus velezensis* **(D)**, and *Bacillus subtilis* strain Bs248 **(E)** and Bs290 **(F)**. P, phosphorus; N, nitrogen; SDM, shoot dry matter; RDM, root dry matter; TDM, total dry matter; CO_2_, respiratory activity; CBM, biomass carbon; and CFU, colony-forming units.

The soil respiratory activity reached the highest value (14.98 mg CO_2_/100 g soil) at a concentration of 1 × 10^8^ conidia ml^–1^ compared with control, 3.50 mg CO_2_ (Figure 6B); and the number of colony-forming units in leaves was higher for all inoculant concentrations compared with control (Figure 7B). For values of colony-forming units in roots, although presenting no interaction, there was a significant effect of the microorganism factor, where *A. brasiliensis* stood out, with 3.92 CFU mL^–1^ (*p* < 0.039, Figure 9A); in addition, a positive correlation (*p* < 0.05) was observed between inoculant concentration and the number of colony-forming units in roots (Figure 10A). *A. brasiliensis* was isolated from the cotton plant, demonstrating that this fungus was probably able to colonize and enter the plant, showing its effects as an endophytic growth-promoting fungus on cotton. *A. brasiliensis* is described as a fast-growing and sporulating species, with characteristics closely related to *Aspergillus niger* (Varga et al., 2007); and *A. sydowii* is described as one of the fungi most commonly found in the soil (Raper and Fennell, 1965; Klich, 2002) and is used in industry for the production of enzymes such as β-glucosidase, α-galactosidase, cellulase, and xylanase (Tian et al., 2016).

For *A. sydowii*, the unfolding of interactions indicates that the shoot phosphorus content presented lower value at a concentration of 1 × 10^8^ conidia ml^–1^ (1.85 g P/kg, Figure 2J) when compared with control (2.17 g P/kg); the soil phosphorus content was lower with 11.68 mg P/dm^3^ at a concentration of 1 × 10^6^ conidia ml^–1^, and control reached 26.91 mg P/dm^3^ (Figure 4C); the nitrogen percentage in soil inoculated with *A. sydowii* at all concentrations was lower than that of control (Figure 5C); the soil respiratory activity was higher (10.43 mg CO_2_/100 soil) with inoculation at a concentration of 1 × 10^8^ conidia ml^–1^ compared with control, which was 3.5 mg CO_2_/100 soil (Figure 6C) and for colony-forming units in leaves, highlighting inoculation of *A. sydowii* at a concentration of 1 × 10^10^ conidia ml^–1^ with 43.00 CFU mL^–1^ compared with control, 1.33 CFU mL^–1^ (Figure 7C).

For *Aspergillus* sp. *versicolor* section, the interaction indicates that the highest nitrogen content in shoot dry matter was obtained at the lowest concentration of 1 × 10^4^ conidia ml^–1^ (24.86 g N/kg; Figure 2D), when compared with control, 20.02 g N/kg; there was a positive correlation (*p* < 0.05, Figure 10C) between inoculum concentration and soluble phosphorus in soil, and the largest amount (62.00 mg P/dm^3^ soil) was obtained at a concentration of 1 × 10^10^ conidia ml^–1^ (Figure 4D) and control only 26.91 mg P/dm^3^ soil; and the soil nitrogen percentage was lower at all concentrations when compared with control (Figure 5D).

For colony-forming units in roots, there was a significant effect (*p* < 0.039, Figure 9A) of the microorganism factor, where *Aspergillus* sp. *versicolor* section stood out from control, with 4.58 CFU mL^–1^, and a positive correlation (*p* < 0.05) was observed between concentration and the number of colony-forming units in roots (Figure 10C). The greatest amount of CFU mL^–1^ in roots and soil was reached when plants were inoculated at maximum concentration (1 × 10^10^ conidia ml^–1^), regardless of fungus used (*A. brasiliensis*, *A. sydowii*, and *Aspergillus* sp. *versicolor* section) (Figures 9B,C).

For *A. brasiliensis* and *A. sydowii*, the increase in inoculum concentration had a positive effect on variable colony-forming units in leaves (Figures 7B,C); however, a concentration of 1 × 10^6^ conidia ml^–1^ of *A. brasiliensis* proved to be appropriate to obtain higher shoot nitrogen contents (Figure 2B), and a concentration of 1 × 10^8^ conidia ml^–1^ of *A. brasiliensis* or *A. sydowii* was suitable for higher respiratory activity values (Figures 6B,C).

The highest inoculant concentrations promoted the highest numbers of CFU mL^–1^ recovered from cotton roots and leaves. Endophytism promotes a more intimate interaction between a microorganism and a host, intensifying the benefits for both (Hardoim et al., 2008; Nadeem et al., 2014; Khan et al., 2015). Interestingly, treatments that presented a greater number of endophytic microorganisms did not necessarily promote greater plant development. Lobo et al. (2019) verified that the treatment that promoted a higher maize yield under field conditions, compared with control, also presented a lower number of recovered CFU mL^–1^. These results suggest that the growth-promoting effect probably depends more on the abilities of microorganisms and the interaction between microorganism and plant than on higher CFU mL^–1^ values.

According to results of the present study, the hypothesis that the highest *A. brasiliensis* and *A. sydowii* concentrations positively affect microorganism colonization can be confirmed. However, this greater colonization did not reflect in greater plant development. These results also show that *A. brasiliensis* and *A. sydowii* are fungi with endophytic capacity in cotton plants. This characteristic in both fungi is an advantage because the endophytic colonization of plant tissues allows the fungus to establish itself inside the organs for some time without causing apparent damage to the host (Petrini, 1991), in addition to protecting plants against eventual colonization and pathogen infection or pest infestation (Bulgarelli et al., 2013). Studies carried out in China have shown that *A. niger* P85 has the ability to solubilize phosphorus, produce indole acetic acid in maize plants, and increase available phosphorus in the soil (Yin et al., 2015); and in Brazil, similar studies have demonstrated the ability of *A. sydowii* and *A. brasiliensis* as phosphorus solubilizers in maize plants (Baron et al., 2018). *A. brasiliensis* and *A. sydowii* have great potential for use in other agricultural crops of great economic importance.

For *Aspergillus* sp. *versicolor* section, increasing inoculum concentration had a positive effect on soil phosphorus concentration and number of colony-forming units in roots (Figures 10B,C); however, a concentration of 1 × 10^4^ conidia ml^–1^ was suitable for cotton plants to show the highest shoot nitrogen content (Figure 2D).

*Aspergillus* sp. *versicolor* section are accepted as distinct species based on molecular and phenotypic differences, are isolated from soil, and adapt to form part of the rhizospheric plant community (Zeljko et al., 2012). *Aspergillus* sp. *versicolor* section are fungi that are part of the microbial community of the rhizosphere of tea plants (Rahi et al., 2009). Similarly, in the present study, *Aspergillus* sp. *versicolor* section showed soil phosphorus solubilization capacity and root colonization. These characteristics are interesting in agriculture because inoculation with higher *Aspergillus* sp. *versicolor* section concentrations could decrease the need for use of mineral fertilizers in the field (Qiao et al., 2019; Caruso et al., 2020) as a consequence of the more efficient use of these fertilizers by plants. Some studies have shown that the association of this fungus with roots promotes abiotic stress tolerance and protection against pathogens (Singh et al., 2012; Begum et al., 2019; Rana et al., 2019).

For *B. velezensis*, the unfolding of interactions indicates that the nitrogen content in shoot dry matter of cotton plants was higher with 22.46 g N/kg at a concentration of 1 × 10^8^ CFU mL^–1^ compared with control, 20.02 g N/kg (Figure 2E); the phosphorus content in the root dry matter and in the soil at all concentrations did not differ from that of control (Figures 3L, 4E); the soil nitrogen percentage was lower at all concentrations compared with that of control (Figure 5E); the respiratory activity was higher at all concentrations when compared with that of control (Figure 6E); the amount of colony-forming units in leaves, roots, and soil was higher at a concentration of 1 × 10^10^ CFU mL^–1^ (34.00, 93.67, and 163.33 CFU mL^–1^, respectively; Figures 8B,F,J); in addition, there was a positive correlation between concentration and colony-forming units in leaves (*p* < 0.05, Figure 10D).

For inoculation of *B. subtilis* Bs248, interaction indicates that the concentration of 1 × 10^10^ CFU mL^–1^ in cotton plants promoted the highest nitrogen content in the root dry matter (12.41 g N/kg) when compared with control (9.35 g N/kg) (Figure 3F); the phosphorus content in the root dry matter was not affected by concentration (Figure 3M); soil phosphorus at a concentration of 1 × 10^10^ CFU mL^–1^ was approximately double (53.15 mg P/dm^3^) that found at concentrations of 1 × 10^4^, 1 × 10^6^, and 1 × 10^8^ CFU mL^–1^ and control (Figure 4F); in addition, there was a positive correlation between variable soil phosphorus and concentration (*p* < 0.05, Figure 10E); soil nitrogen percentage was lower, and the respiratory activity was higher when *B. subtilis* Bs248 was inoculated at any concentration (Figures 5F, 6F). The number of colony-forming units in leaves was higher when inoculum was applied at concentrations of 1 × 10^8^ and 1 × 10^10^ CFU mL^–1^ (Figure 8C); the number of colony-forming units in roots was greater when inoculum was applied at a concentration of 1 × 10^6^ CFU mL^–1^ (Figure 8G), and the number of colony-forming units in soil was greater at concentrations of 1 × 10^6^ and 1 × 10^10^ CFU mL^–1^ (Figure 8K).

For *B. subtilis* Bs290, interaction indicates that the inoculation of cotton plants at a concentration of 1 × 10^4^ CFU mL^–1^ had the lowest nitrogen percentage, 5.97%, when compared with control, which reached 8.77% (Figure 5G), and a smaller amount of colony-forming units in leaves with 5.00 CFU mL^–1^, when compared with control of 18.00 CFU mL^–1^ (Figure 8D); the number of colony-forming units in roots was higher, with 15.67 and 10.67 CFU mL^–1^, when the microorganism was inoculated at concentrations of 1 × 10^6^ and 1 × 10^8^ CFU mL^–1^, respectively (Figure 8H); and the number of colony-forming units in soil was higher, with 192.67 and 194.33 CFU mL^–1^, when inoculated at concentrations of 1 × 10^8^ and 1 × 10^10^ CFU mL^–1^, respectively (Figure 8L). Additionally, a positive correlation was observed between concentration and respiratory activity (*p* < 0.05, Figure 10F).

Most *Bacillus* species are considered plant growth-promoting rhizobacteria and have the ability to colonize roots, improve nutrient availability, reduce abiotic stress, and produce a wide range of biologically active secondary metabolites that can inhibit the growth of pathogens (Ongena and Jacques, 2008; Lugtenberg and Kamilova, 2009; Bhattacharyya and Jha, 2012; Sivasakthi et al., 2014). The increase in inoculum concentration had a positive effect on variable colony-forming units in leaves for *B. velezensis*, soil phosphorus for *B. subtilis* Bs248, and a respiratory activity for *B. subtilis* Bs290.

*Bacillus velezensis* was previously grouped with *B. subtilis* and *Bacillus amyloliquefaciens*, and in recent years, several isolates of this bacterium have received attention due to their potential in disease control (Fan et al., 2017; Adeniji et al., 2019). Previous studies have determined that *B. velezensis* has the ability to produce indole acetic acid in pepper plants applied at a concentration of 1 × 10^8^ CFU mL^–1^ (Zhang et al., 2019); in addition, it has been shown that metabolites produced have an antagonistic activity against bacterial and fungal pathogens under laboratory and greenhouse conditions in tomato crops (Cao et al., 2018). In the present study, *B. velezensis* showed the ability to colonize cotton leaves as the inoculum concentration increases. These results demonstrate that *B. velezensis* is an endophytic bacterium with capacity to promote growth through nitrogen content in shoot dry matter; in addition, results of colony-forming units in leaves suggest that *B. velezensis* has potential to inhibit the growth of pathogens in cotton plants.

On the other hand, studies have demonstrated the ability of *B. subtilis* to solubilize phosphate, produce indole acetic acid and siderophores, and increase dry weight in maize and sorghum (Aquino et al., 2019), okra, spinach, and tomato plants, in addition to presenting antagonistic action against *Rhizoctonia solani* (Adesemoye et al., 2009). Regarding colonization, studies carried out with cucumber and tomato plants inoculated with *B. subtilis* at concentrations of 10^5^ and 10^6^ CFU mL^–1^ of root were enough for the microorganism to be able to colonize and survive in the rhizosphere. Thus, in addition to protecting plants by suppressing *Fusarium oxysporum* from cucumber, *B. subtilis* had an antagonistic effect against *Pseudomonas syringae* after root colonization in tomato plants (Cao et al., 2011; Chen et al., 2013). In the present study, *B. subtilis* strains have shown a correlation between soil phosphorus content and respiratory activity. These results suggest that to improve phosphorus solubilization and respiration in the soil, it is necessary to increase inoculum concentration.

On the other hand, studies have shown that the long-term continuous use of inoculants influences the quantity and quality of microorganisms present in the soil rhizosphere, but this depends on conditions such as organic matter, availability of nutrients (such as phosphorus), and type of soil (Gnankambary et al., 2008; Angelina et al., 2020). Furthermore, it is important to consider that the composition of the soil community is largely influenced by environmental variability and the microbial community present in the soil (Xun et al., 2015).

As one of the most important and essential macronutrients in addition to nitrogen, phosphorus is important for plant development, but it is the nutrient element least mobile in plant and soil. Globally, P is extracted from geological sediments and added to agricultural soils in order to meet critical plant requirements for agronomic productivity. Phosphorus is present in soil in the organic and inorganic forms. The various inorganic forms of the element in the soil are salts with calcium, iron, and aluminum, while the organic forms come from decomposing vegetation and microbial residues. There is great diversity of plant microbiomes (epiphytic, endophytic, and rhizospheric) and soil microbiomes that have the ability to solubilize insoluble P and make it available for plants. The main solubilization mechanism of inorganic P is by the production of organic acids, which lower soil pH, or by the production of acids and alkaline phosphatases, which cause the mineralization of organic P. P-solubilizing and P-mobilizing microorganisms belong to all three domains: archaea, bacteria, and eukarya. Strains belonging to genera *Arthrobacter*, *Bacillus*, *Burkholderia*, *Natrinema*, *Pseudomonas*, *Rhizobium*, *Serratia*, and *Aspergillus* have been reported as efficient and potential P solubilizers. The use of P solubilizers, alone or in combination with another plant growth-promoting microbe as an ecological microbial consortium, could increase P uptake by plants, increasing their yields for agricultural and environmental sustainability (Kour et al., 2021). However, results have shown that for some treatments, phosphorus concentrations in soil and roots decreased. Factors such as mineral concentration, temperature, and availability of carbon and nitrogen (N) sources can affect the phosphorus solubilization potential of these microorganisms, and these results suggest that there was greater solubilization and absorption of phosphorus from the soil by plants and greater translocation to shoots.

For the field phase, *A. sydowii* was selected for presenting abilities to promote a positive effect on variables shoot and total dry matter, soil respiratory activity, and colony-forming units in leaves and roots; *Aspergillus* sp. *versicolor* section were selected for presenting the ability to promote positive effects on variables shoot and total dry matter, nitrogen content in shoot dry matter, colony-forming units in roots and soil phosphorus; *B. velezensis* (Bv188) was selected for presenting the ability and promoting positive effects on variables nitrogen content in shoot dry matter, respiratory activity, colony-forming units in leaves, roots, and soil; and *B. subtilis* 248 was selected for presenting the ability to promote positive effects on variables root nitrogen content, soil phosphorus, respiratory activity in soil, and colony-forming units in leaves, roots, and soil.

### Experiment 2: Determination of the Effect of Inoculation of Microorganisms on Cotton Plants Under Field Conditions

**FIGURE 11:**
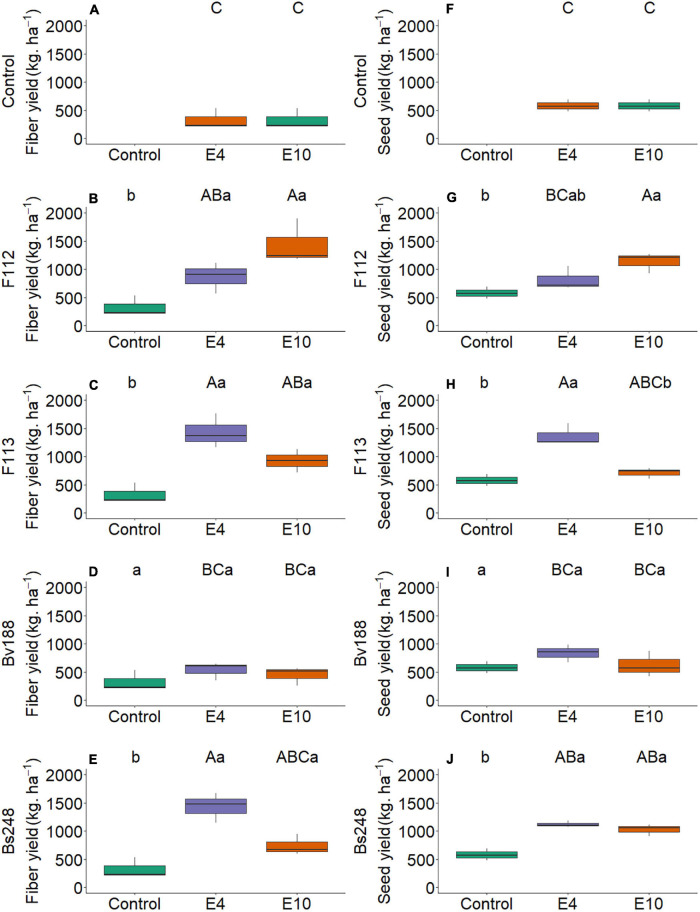
Boxplots (median and quartiles) of fiber **(A–E)** and seed **(F–J)** cotton yield inoculated with plant growth-promoting microorganisms in two concentrations. Different lowercase letters in a row and uppercase letters in a column indicate statistical difference between means (Tukey, *p* < 0.05). F112, *Aspergillus sydowii*; F113, *Aspergillus* sp.; Bv188, *Bacillus velezensis* strain Bv188; Bs248, *Bacillus subtilis* strain Bs248; E4, 1 × 10^4^; E10, 1 × 10^10^ conidia or CFU mL^−1^; Ctrl, control; and CFU, colony-forming units.

Regarding field yield, there was no interaction of concentration factor and microorganism factor on variables fiber yield (Figures 11A–E) and seed yield, except for *Aspergillus* sp. *versicolor* section (F113), which presented the lowest yield for a concentration of 1 × 10^10^ CFU mL^–1^ compared with a concentration of 1 × 10^4^ CFU mL^–1^ (Figure 11H). Fiber yield in cotton plants inoculated with *B. velezensis*, *B. subtilis* 248, *A. sydowii*, and *Aspergillus* sp. *versicolor* section were superior to control, which had 326.94 kg/ha (Figures 11A–F). Inoculation of *A. sydowii* at a concentration of 1 × 10^10^ conidia ml^–1^ and *Aspergillus* sp. *versicolor* section at a concentration of 1 × 10^4^ conidia ml^–1^ had the highest seed yield, with 1,131.14 and 1,364.96 kg/ha, respectively (Figures 11G,H). Inoculation with *B. velezensis* at a concentration of 1 × 10^4^ and 10^10^ CFU mL^–1^ showed no differences when compared with that with control (Figure 11I). Inoculation with *B. subtilis* Bs248 showed no differences between concentrations of 1 × 10^4^ and 1 × 10^10^ CFU mL^–1^, reaching values of 1,118.54 and 1,024.68, respectively (Figure 11J).

For *A. sydowii* and *B. subtilis* Bs248, the hypothesis that fiber and seed yield at concentrations of 1 × 10^4^ or 1 × 10^10^ CFU mL^−1^ are similar is confirmed. Thus, the results of the present study demonstrate that there is no effect of concentration on cotton seed and fiber yield when inoculated with *A. sydowii* and *B. subtilis* Bs248 and that there is no effect of concentration on cotton seed yield when inoculated with *Aspergillus* sp. *versicolor* section.

Yield studies performed with *A. sydowii* and *Aspergillus* sp. *versicolor* section in cotton are scarce in scientific literature; for example, studies carried out on chickpea plants have shown the ability of fungi *Aspergillus awamori* and *Penicillium citrinum* inoculated at a concentration of 1 × 10^6^ spores/ml to increase seed weight by approximately twice (Mittal et al., 2008). In addition, *A. niger*, *Aspergillus fumigatus*, and *Penicillium pinophilum* inoculated on wheat and fava beans at a concentration of 2 × 10^9^ spores/ml^–1^ increased yield by 28.9–32.8% and 14.7–29.4%, respectively (Abdul Wahid and Mehana, 2000). Likewise, phosphorus uptake by both cultures increased due to inoculation with tested fungi. Other studies include arbuscular mycorrhizal fungi in maize plants using concentrations of 1 × 10^3^ spores/ml where, in addition to increasing yield by 80%, these fungi are capable of inducing resistance against pathogenic *A. niger* strains (Molo et al., 2019).

For plant-growth promoting bacteria, Tripti et al. (2017) observed increase in the amount of fruits on tomato plants inoculated with *Bacillus* sp. strain A30 and *Burkholderia* sp. strain L2 at a concentration of 10^10^ CFU mL^–1^. Furthermore, inoculation with *A. brasiliensis* Ab-V5 and *B. subtilis* strain CCTB04 at a concentration of 1 × 10^8^ CFU mL^–1^ positively affected corn yield by 39.5 and 29.1%, respectively (Pereira et al., 2020).

Microorganisms *A. sydowii*, *Aspergillus* sp. *versicolor* section, and *B. subtilis* Bs248 used at concentrations of 1 × 10^4^ and 1 × 10^10^ conidia or CFU mL^–1^ in the field phase allow achieving similar results in cotton fiber and seed yield. These results show that lower inoculant concentrations could be used with no damage to plant growth efficiency promoted by the microbial isolate.

## Conclusion

The parameters that were favored by the highest inoculant concentrations were soil respiratory activity, phosphorus in root dry matter, nitrogen in shoot dry matter, and number of colony-forming units in roots and leaves. Concentrations did not affect nitrogen in root dry matter, phosphorus in shoot dry matter, and microbial biomass carbon. However, other factors such as nitrogen and phosphorus contents in the soil, except for *Aspergillus* sp. *versicolor* section, were negatively affected with the highest inoculant concentrations. Interestingly, inoculant concentrations did not affect cotton fiber or seed yield.

The present study brings results that help in a better understanding of the effect of concentrations of fungi- and bacteria-based inoculants on the biometric parameters of plants, on microbial activities and soil fertility, on the nutritional status of plants, and on cotton crop productivity.

## Data Availability Statement

The raw data supporting the conclusions of this article will be made available by the authors, without undue reservation.

## Author Contributions

All authors listed have made a substantial, direct and intellectual contribution to the work, and approved it for publication.

## Conflict of Interest

The authors declare that the research was conducted in the absence of any commercial or financial relationships that could be construed as a potential conflict of interest.

## Publisher’s Note

All claims expressed in this article are solely those of the authors and do not necessarily represent those of their affiliated organizations, or those of the publisher, the editors and the reviewers. Any product that may be evaluated in this article, or claim that may be made by its manufacturer, is not guaranteed or endorsed by the publisher.
